# Primary orbital ganglioneuroblastoma in a child: A case report

**DOI:** 10.1097/MD.0000000000022922

**Published:** 2020-11-06

**Authors:** Yi Zhang, Weimin He

**Affiliations:** Department of Ophthalmology, West China Hospital of Sichuan University, No. 37 Guoxue Xiang, Wuhou District, Chengdu, Sichuan Province, China.

**Keywords:** ganglion cell, ganglioneuroblastoma, neuroblast

## Abstract

**Rationale:**

Ganglioneuroblastoma (GNB) is a transitional tumor of sympathetic origin that has never been described as primarily involving the orbit. Herein we report an extremely rare case of GNB with primary orbital involvement and its treatment strategies.

**Patient concerns:**

A 9-year-old girl presented with progressive and recurring right orbital mass for 2 years.

**Diagnosis:**

Computed tomography (CT) showed a well-defined, well-circumscribed, and homogeneous extraconal soft tissue mass occupying most of the right superior orbital area. Magnetic resonance imaging (MRI) revealed that there was a neoplasm of the right superior orbit molding around the globe with long T1 and T2 signals, and contrast-enhanced MR image showed a heterogeneous enhancement of the mass. Histopathologic examinations were performed after surgery and the characteristics were consistent with a diagnosis of GNB.

**Interventions:**

Surgery was performed and the mass was completely resected.

**Outcomes:**

Postoperatively, the patient was on a regular follow-up for 19 months and so far, has had no orbital mass recurrence.

**Lessons:**

Herein we present a rare case of GNB primarily involving the orbit, and the findings showed that GNB could originate from the orbit. The patient underwent surgical tumor resection. The histopathological and immunohistochemical features were consistent with the diagnosis of GNB. For this case, there was no recurrence for 19 months after complete surgical excision of the tumor; however, a regular long-term follow-up is required.

## 1 Introduction

Neuroblastic tumors (NTs) are one of the most common solid tumors in children, which are derived from primitive cells of the sympathetic nervous system and arise from the neural crest.^[1]^ Including a group of diseases, NTs range from immature, undifferentiated to mature, and differentiated tumors, which are named neuroblastoma, ganglioneuroblastoma, and ganglioneuroma.^[2]^ The 2 extremes of NTs represent the most malignant neuroblastomas and the most benign ganglioneuromas. In between lie GNBs, which are characterized by a mixture of cells ranging from primitive neuroblasts to well-differentiated ganglion cells within neurofibromatous tissues.^[3]^ GNBs can be further sub-classified into 2 types: nodular and intermixed.^[3]^ It is a transitional tumor of sympathetic origin, which has not yet been described as primarily involving the orbit. The most common sites of GNB origin are the adrenal medulla, extra-adrenal retroperitoneum, and posterior mediastinum.^[4]^ Herein we report a rare case of a 9-year-old girl with a primary GNB of the orbit who responded favorably to surgical treatment.

## 2 Case presentation

**Figure 1:**
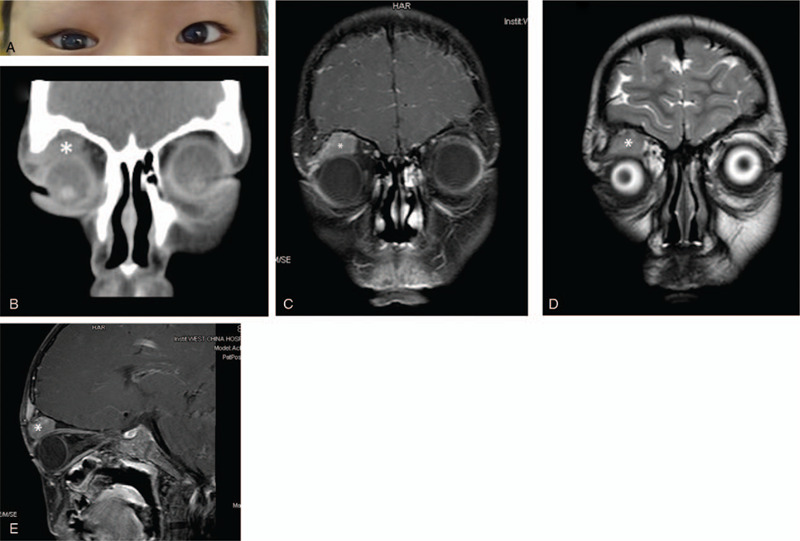
A. The appearance of the eyes with right orbital tumors. B. CT scans revealing the location of the orbital tumor (white asterisk) in the superior orbit. C and D. Coronal T1-weighted MR image of the orbital tumor (white asterisk) (C) and Coronal T2-weighted MR image of the orbital tumor (white asterisk) (D). E. Sagittal T1-weighted MR image of the orbital tumor (white asterisk).

This case report was approved by the Ethics Committee, and written informed consent was obtained. A 9-year-old girl was referred to our hospital in December 2018 because she presented with progressive and recurring right orbital masses for 2 years (Fig. 1A). Five years ago, the patient underwent a surgical tumor excision of the right orbital region in another hospital because of progressive right orbital masses for 1 year. The histopathological diagnosis was ganglioneuroma. After the surgery, the patient did not receive any other treatment, including chemotherapy. No prior history of ocular discharge or ocular trauma was recorded. Her family history was unremarkable. Proptosis of the right ocular globe and palpable superior orbital masses were the main clinical findings. This neither had Horner syndrome (proptosis, pupillary constriction, and ipsilateral facial anhidrosis) or opsoclonus.

**Figure 2:**
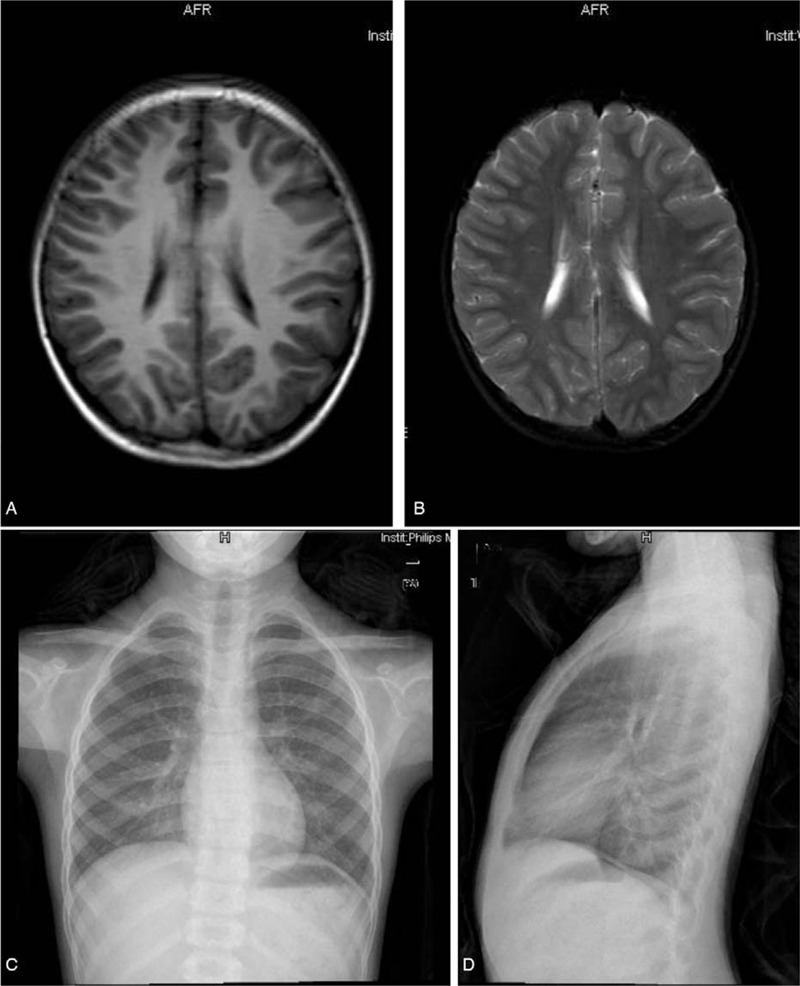
Axial T1-weighted and T2-weighted MR image of head (A-B). Frontal chest radiograph (C) and Lateral chest radiograph (D).

Computed tomography (CT) and magnetic resonance imaging (MRI) scans of her orbit were performed (Fig. 1B-E). CT showed a well-defined, well-circumscribed, and homogeneous extraconal soft tissue mass occupying most of the superior orbital area, measuring 2.2 cm × 1.8 cm, with evidence of bony destruction but no calcification. MRI revealed that there was a neoplasm of the right superior orbit that molding around the globe, measuring 2.1 cm × 1.4 cm × 1.9 cm with long T1 and T2 signals, and a contrast-enhanced MR image showed that there was a heterogeneous enhancement of the mass. The mass caused an inferotemporal right globe displacement. Head MRI and chest X-ray results were normal (Fig. 2A-D).

**Figure 3:**
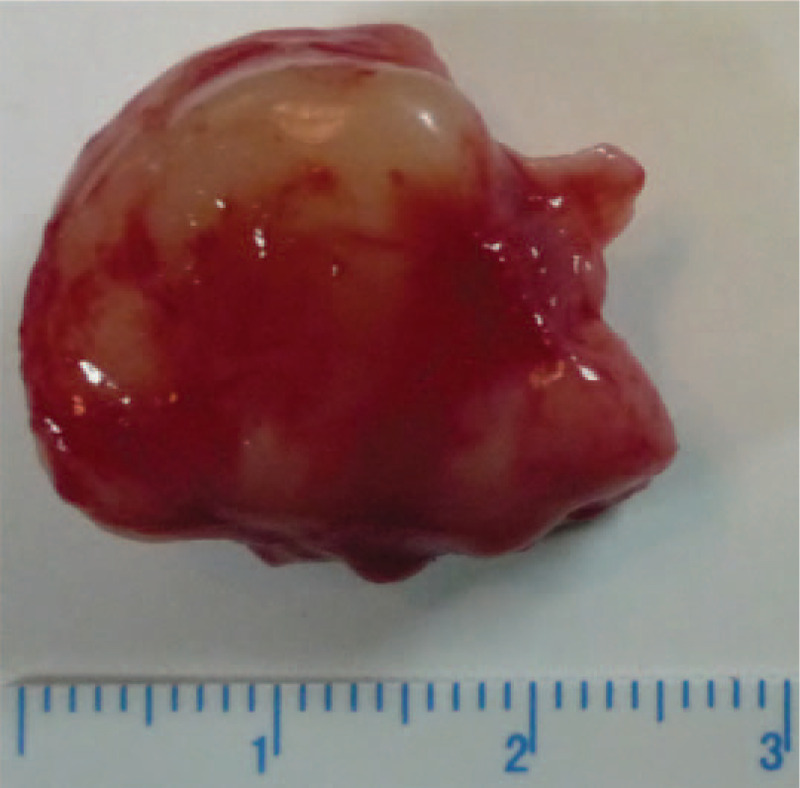
Resection of tumor during surgery and it is in the superior orbit.

**Figure 4:**
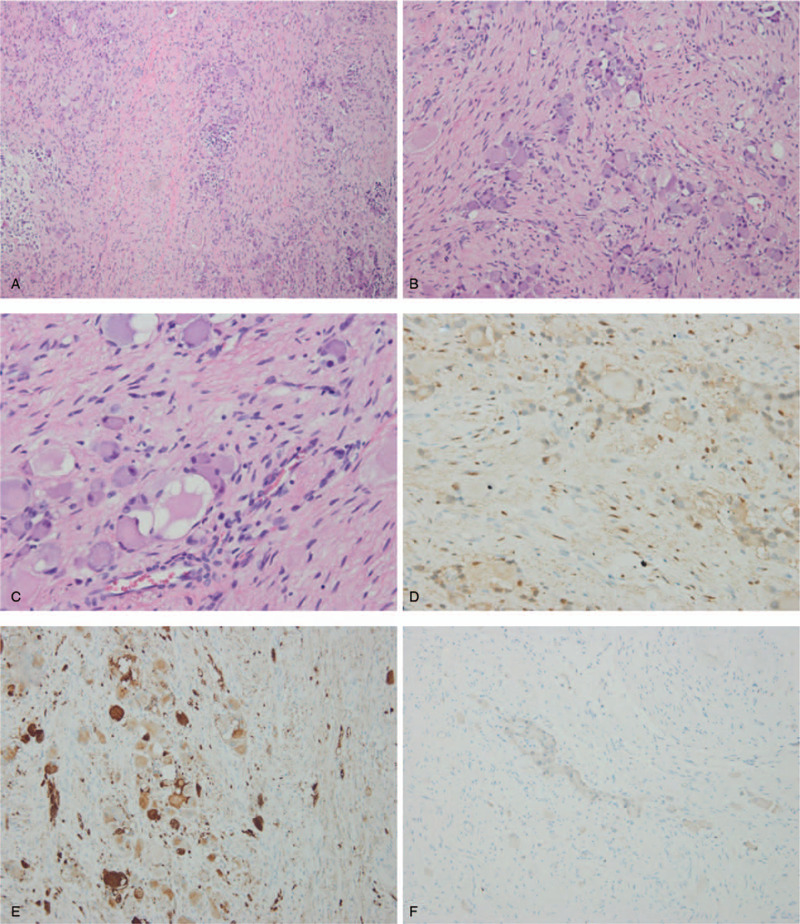
A-C. Hematoxylin–eosin staining showing revealed scattered nests of mature ganglion cells within a matrix of proliferating spindle cells (representing Schwann cells) and neuroblastoma [original magnification ^∗^100 (A), ^∗^200 (B), ^∗^400 (C)]. D. Immunohistochemical staining showed positive staining for S–100 (^∗^400). E. Immunohistochemical staining showed positive staining for Syn (^∗^200). F. Immunohistochemical staining showed positive staining for MIB-1 (^∗^200).

Surgery was performed under general anesthesia through an anterior orbitotomy via a lid crease incision. The incision was below the arch of the eyebrow and on the surface of the mass. Orbital exploration revealed a circumscribed lesion with red–yellow surface without necrosis (Fig. 3). The mass was carefully separated from the superior rectus and optic nerves, and then completely resected. Incisional biopsy was performed postoperatively. Gross examination showed that the dimension of the mass was 2.2 cm × 2 cm × 1.5 cm, and hematoxylin–eosin staining revealed scattered nests of mature ganglion cells within a matrix of proliferating spindle cells and neuroblastoma (Fig. 4A-C). Immunohistochemical staining showed positive staining for S100 and Syn (Fig. 4D-E), but negative for LCA and CR. The Ki67 labeling index was 1% (Fig. 4F). These characteristics were consistent with the diagnosis of GNB.

Postoperatively, there was no injury to her visual acuity and no limitation of eyeball movements of the right eye. The patient had a smooth course, and was on regular follow-up for 19 months and so far, has had no orbital mass recurrence.

## 3 Discussion and conclusion

NTs are a group of tumors that can be seen in the neck, posterior mediastinum, adrenal gland, retroperitoneum, and pelvis.^[4]^ NTs differ in their degrees of cellular and extracellular maturation; immature tumors tend to be aggressive and occur in younger patients, whereas mature tumors occur in older children and tend to behave in a benign manner.^[4]^ GNB has an intermediate malignant potential, as it is composed of both mature gangliocytes and immature neuroblasts. The presence of immature tissue in GNB indicates malignancy or potentially malignant behaviors.^[5]^ As 1 type of NT, GNB also arises from the sympathetic chain. The most common sites of origin of GNB, in decreasing order, are the adrenal medulla (35% of cases), extra-adrenal retroperitoneum (30%–35%), and posterior mediastinum (20%); the less common sites are the neck (1%–5%) and pelvis (2%–3%).^[4,6]^ Case reports on GNBs are extremely rare, especially with orbital involvement. Herein we report a very rare case of GNB with primary orbital involvement. Most GNBs in other sites excrete large amounts of vanillylmandelic acid. Due to the expression of vasoactive intestinal peptide, a syndrome of failure to thrive and intractable watery diarrhea may occur with these tumors.^[7]^ In our case, the patient did not have any other symptoms besides the orbital masses and had normal growth. In addition, the body check and imaging showed no mass in other sites. Based on these findings, we could conclude that this was a rare case of GNB with primary orbital involvement. However, this patient still needs longer follow-up to ensure that there is no metastasis.

Because GNBs arise in various locations, they have varied appearances and growth patterns. The diagnosis mainly relies on clinical symptoms, imaging, histopathologic examination, and immunohistochemical staining.^[8]^ For imaging GNB, MRI and CT scanning are preferred methods. On MRI, GNB is typically heterogeneous, variably enhanced, and of relatively low signal intensity on T1-weighted images and high signal intensity on T2-weighted images.^[4]^ As for this case, it showed atypical images, and the diagnosis mainly relied on histopathologic examination and immunohistochemical staining. In this case, the mass contained both mature ganglion cells and neuroblastoma. H-E staining showed that neuroblasts were immature and undifferentiated sympathetic cells, which were small, rounded in contour, showed little cytoplasm, and possessed darker nuclei and smaller indistinct nucleoli, while ganglion cells were fully mature cells with abundant cytoplasm, rounded contour, and large nuclei with distinct and prominent nucleoli.^[4]^ For immunohistochemical staining, Syn and S-100 are generally positive in Ganglioneuroblastoma.^[9]^ In this case, the histopathologic examination and immunohistochemical staining characteristics were consistent with that of ganglioneuroblastoma.

The treatment choice for GNBs mainly relies on the stage of the disease. According to the International Neuroblastoma Staging System (INSS),^[10]^ this case was classified as stage 1. GNB is widely seen as a malignant entity and is treated with multimodal therapy. Complete surgical excision of the tumor is one of the therapeutic options. Decarolis et al^[11]^ showed no disease progression after complete resection and subtotal resection with residuals smaller than 2 cm. Other studies also presented the proposal that resection did not have to be radical for the treatment of GNB, especially when considering surgical complications.^[12,13]^ However, immature areas of the tumor should be sufficiently resected in the initial surgery since there is a possibility that the large tumor masses contain immature components, which may be sources of disease progression. Chemotherapy seems not to be indicated for patients with localized GNBs and the cytotoxic treatment has no substantial effect, whereas surgery alone is sufficient for the treatment of GNB and does not need to be radical if only minor residuals are left.^[11]^ As for this case, the patients first surgery might have been insufficient to remove the mass and might cause tumor recurrence. However, we could not rule out the possibility that we were dealing with a new tumor.

GNB, histologically containing primitive neuroblasts, is therefore considered malignant or potentially malignant. The prognosis is uncertain, as they can undergo spontaneous regression or spontaneous differentiation to benign neoplasms or exhibit malignant behaviors, especially in older children.^[14]^ As reported in other cases, GNB originated from other sites with orbital metastases, and the prognosis was reportedly poor, with one large series of findings with only an 11.2% survival rate.^[14]^ However, in our case, the patient had no sign of orbital mass recurrence and metastasis until now. We suppose that this was because it was a localized primary orbital involved GNB without tumor metastasis, which could be completely resected and the Ki67 labeling index was 1%. However, it should be emphasized that regular long-term follow-up is warranted.

In summary, we present a rare case of GNB primarily involving the orbit. The patient underwent surgical tumor excision, and the histopathological features were consistent with the diagnosis of GNB. For the diagnosis of this case, imaging of the mass might not show typical images, and the diagnosis mainly relied on histopathologic examination and immunohistochemical staining. For this case, there was no recurrence for 19 months after complete surgical excision of the tumor, but the patient should undergo a regular long-term follow-up.

## Author contributions

YZ collected the data of the patient, consulted literatures, dealt with the Figs and wrote the manuscript; WMH was the Consultant in charge for the case, established the diagnosis, performed the surgery and approved the submitted version. All authors read and approved the final manuscript.

**Data curation:** Yi Zhang.

**Formal analysis:** Yi Zhang.

**Project administration:** Weimin He.

**Supervision:** Weimin He.

**Writing – original draft:** Yi Zhang.

**Writing – review & editing:** Weimin He.
